# When to Stop Tyrosine Kinase Inhibitors for the Treatment of Chronic Myeloid Leukemia

**DOI:** 10.1007/s11864-018-0532-2

**Published:** 2018-03-08

**Authors:** Pierre Laneuville

**Affiliations:** McGill University Health Centre, 1001 Decarie Blvd, Room D02.7722, Montreal, Quebec H4A 3J1 Canada

**Keywords:** Leukemia, Chronic myelogenous leukemia, Chronic myeloid leukemia, CML, Treatment-free remission, TFR

## Abstract

Strict criteria for when to stop tyrosine kinase inhibitor (TKI) therapy in clinical practice are not easily defined without an agreement on what probability of achieving a treatment-free remission (TFR) constitutes a medically acceptable standard and consideration of the potential medical risks of continued TKI therapy and/or patient preferences. Patients in sustained deep molecular response (DMR) have no significant chronic myelogenous leukemia-related risk of progression and death, and thus, safety is of paramount importance. Patients with prior history of advanced disease, additional chromosomal abnormalities (ACA), atypical transcripts, TKI resistance, high Sokal score, or who cannot be relied upon to come for regular molecular monitoring should generally be excluded from TKI cessation in clinical practice. Similarly, stopping TKIs should not be attempted without the availability of standardized *BCR-ABL1* testing with a sensitivity of at least MR4.5 and a turnaround time of less than 4 weeks. Prior TKI therapy of 5 years and stable MR4.0 of 2 years or more constitutes reasonable minimal criteria for stopping TKIs with approximately a 50% chance of success. The risk of morbidity with continued TKI therapy and patient preferences need to be considered to determine to what extent these minimal criteria should be exceeded and at what threshold to re-initiate therapy whether on the loss of major molecular response or at a lower molecular endpoint.

## Introduction

The outcome of chronic myelogenous leukemia (CML) patients presenting in the chronic phase has changed dramatically since the introduction of tyrosine kinase inhibitor (TKI) therapy with imatinib in 2001 and second-generation TKIs in 2007. With the availability of TKI therapy, standardized molecular monitoring, and the adoption of response-adapted intervention for patients who “fail” to respond adequately, as defined in evidence-based international guidelines[1, 2], patients with this historically fatal disease now have survival approaching that of the normal population [3]. While it was initially believed that TKI therapy would need to be continued indefinitely, it is now well accepted that a subgroup of patients who achieve a deep and sustained molecular response (DMR) can successfully discontinue TKI therapy and maintain a treatment-free remission (TFR). This was first demonstrated in the STIM1 trial [4] following demonstrated feasibility in a smaller study (STIM-Pilot) [5]. Discontinuation of first-line imatinib in patients who maintained a state of undetectable molecular residual disease (UMRD) for at least 2 years, measured by quantitative real-time reverse transcriptase polymerase chain reaction (qRT-PCR) with a sensitivity of 0.0032% (− 4.5 logs) on the International Scale (IS), UMRD4.5, the molecular relapse-free survival after 60 months was 36% [4]. This sets a precedent for a growing list of TKI discontinuation trials with minimal criteria for the necessary DMR to achieve varying from major molecular response (MMR) to UMRD5.0, sustained for a minimum of 1 to 2 years, and different criteria for the reinstatement of treatment ranging from molecular relapse to the loss of MMR. Variable rates of TFR have been reported with the majority falling in the range of 40 to 60% with success or failure occurring in the first 6 months in the majority of patients. The rate of TFR is strongly influenced by how it is defined as first shown in the A-STIM trial where the estimated rate of TFR increased from 46 to 64% at 2 years using STIM1 versus the loss of MMR as criteria [6]. Results from “real-world” studies are in general agreement with those from prospective clinical trials and have confirmed the importance of maintaining MR4.0 for at least 2 years to ensure a reasonable chance of success. Discontinuation of TKI therapy in the clinical trial setting appears to be safe with the majority of patients who fail to maintain a TFR regaining a DMR after a few months of restarting TKI therapy. Only a single patient has died to date after transforming to advanced phase disease in more than 2500 patients reported. The heterogeneity of trial criteria and results raises several challenges to define criteria for when it is appropriate and safe to stop TKI therapy in general clinical practice. This review highlights some of these challenges.

## TKI discontinuation trials and retrospective series

There is a growing list of prospective TKI discontinuation trials which have been published [4–13] or presented at international meetings [14–24], as well as reports of “real-world” retrospective series of CML patients who have stopped TKI therapy [25–28]. Some key characteristics of these studies (listed in alphabetical order) are given in Table 1 including the number of patients, prior TKI history, median duration of TKI therapy, definition of DMR before discontinuation, the median duration of DMR prior to discontinuation, molecular thresholds used for defining the loss of TFR and the re-initiation of therapy, median follow-up of patients after discontinuation, and the time after discontinuation when the rate of TFR was estimated. The design of the majority of prospective longitudinal studies was inspired by the STIM1 trial by defining minimal criteria for total duration of TKI exposure, DMR and its duration prior to TKI discontinuation, and the molecular endpoint defining TFR failure and re-initiation of TKI therapy. The criteria for DMR range from MR4.0 to UMRD of varying sensitivity and the endpoint for defining the loss of TFR and the indication for retreatment ranges from the loss of UMRD to the loss of MMR. The majority of patients studied to date stopped TKI therapy after first-line imatinib. Several studies included a period of consolidation with dasatinib [7, 19, 23] or nilotinib [16, 17, 22, 24] before TKI discontinuation. ENESTfreedom is the only study in which TKI discontinuation was done in a group of patients who were all uniformly treated in first line with the same second-generation TKI (nilotinib) [22]. Overall, estimates of TFR at 1 year or later range from 33 to 77% with the majority falling between 40 and 60%. The inclusion or not of patients with prior interferon therapy, TKI resistance, or advanced phase disease (i.e., CML AP/BC) introduced additional sources of variability across study reports.

**Table 1:** Summary of TKI discontinuation trials and retrospective series

Study	# Pts	1st-line TKI	2nd-line/consolidation TKI	Median duration TKI (years)	Stable DMR at STOP	Median duration DMR (years)	Retreatment criteria	Follow-up (years)	Time TFR (years)	Rate TFR (%)
A-STIM [6]	80	I (100%)		6.58	UMRD	3.42	> MMR	2.58	2	64
DADI [7]	63	I (100%)	D (100%)	6.83	0.0069%^IS^	NR	> 0.0069%^IS^	1.67	1	48
DASFREE [23]	84	I (85%),D	D (100%)	5.91	MR4.5	NR	> MMR	NR	1	49
Destiny* [15, 29]	117	I (84%), D (8%), N (4%)		6.80	MR4.0*	NR	> MMR	NR	2	77
D-STOP [19]	54	I (61%), D (39%)	D (100%)	7.66	UMRD	4.25	> MR4.0	1.5	1	62.9
ENESTfreedom [22]	190	N (100%)	N (100%)	3.58	MR4.5	2.52	> MMR	NR	1.85	48.9
ENESTop [16]	126	I (100%)	N (100%)	7.3	MR4.5	3.65	> MR4 × 2, > MMR × 1	1.9	1.85	53.2
Euro-Ski [21]	750	I (94%), N/D	15% D/N/I	7.58	MR4.0	2.98	> MMR	0.83	2	51
Ginema [26]	293	I (72%), N (20%), D (8%)		6.42	MR4.0	3.83	Variable	2.83	1	68
Hovon [12]	15	I (100%)		8.17	MR4.5	NR	> 1 log/> MMR	3.6	2	33
ISAV [9]	112	I (100%)		8.59	UMRD	2.14	> UMRD ×2, > MMR	1.8	3	51.9
Japan [28]	43	I (100%)		3.77	UMRD	2.28	> MMR × 2	1.87	5	47
Keio [20]	53	I (91%), N (8%), D (1%)		8.16	UMRD	3.17	> 100 copies *BCRABL*	NR	2	52.8
KID [8]	90	I (100%)		6.73	UMRD	3.32	> MMR × 2	2.22	2	58.5
Korea [27]	24	I (67%), D (21%), B (12%)		6.42	UMRD	4.16	> MMR	3.04	2	59.7
LAST [14]	173	I (60%), N (23%), D (15%), B (2%)		6.58	MR4.0	NR	> MMR	1.025	1	60
MDA** [25]	27	I (77%), D (11%), N (6%), B (6%)		8.0	UMRD	5.25	> UMRD	1.33	1.5	59
NILst [17]	87	I/N	N (100%)	8.6	MR4.5	2–12 Y	> MR4.5 × 2	1.11	1	58.9
STAT2*** [24]	73	I/N	N (100%)	8.52	MR4.5	2^&^, 2.58^&&^	> MR4.5 × 2	NR	1	67.9
STIM1 [4]	100	I (100%)		4.9	UMRD	3.03	> UMRD × 2, > MMR	6.42	5	38
STIM123 [11]	68	I (100%)		8.125	MR4.5	4.5	> MMR	NR	1	67.6
STIM-Pilot [5]	12	I (100%)		3.75	UMRD	2.67	> UMRD × 2	1.5	1.5	50
STOP 2G-TKI [10]	60	D/N 1^st^L 13.3%, 2^nd^L 66.7%, 3^rd^L 20%		6.3	UMRD	2.42	> MMR	3.92	4	53.6
TRAD [18]	123	I (100%)		9.16	MR4.5	NR	> MR4 × 2, > MMR	NR	1	57.5
Twister [13]	40	I (100%)		5.92	UMRD	2.5	> UMRD × 2, > MMR	3.5	2	47.1

*# Pts* number of patients, *TKI* tyrosine kinase inhibitor, *DMR* deep molecular response, *TFR* treatment-free remission, *I* imatinib, *D* dasatinib, *N* nilotinib, *B* bosutinib, *UMRD* undetectable molecular residual disease, *MR* molecular response, *MMR* major molecular response, *IS* international standard, *NR* not reported

*MR4 subgroup

**UMDR subgroup

***Median duration TFR from weighted average of SG1^&^ and SG2^&&^ patient groups

## Study limitations

Studies conducted to date suffer from several limitations. First, a significant number remain unpublished including the largest and perhaps most influential, EURO-Ski. Second, all are non-randomized except for the HOVON trial, a small study comparing patients in DMR randomized to continue imatinib or stop therapy. The absence of randomization complicates the interpretation of many studies, for instance the value of consolidation with a second-generation TKI before discontinuation. Patient attitudes and perceptions regarding treatment cessation have a strong influence regarding their participation potentially introducing selection biases of importance to achieving a TFR [30–33]. Trials with similar minimal criteria for discontinuation may include groups of patients that exceed such criteria by significantly different margins, for instance trials recruited in large measure from a pre-existing pool of patients in DMR. Since TKI therapy entered routine clinical practice at a fixed point in time, this could have the effect of biasing trials that opened later to include patients with a greater total exposure to TKIs and duration of DMR than those that opened earlier. Similarly, while *BCR-ABL1* is a continuous variable, assigning patients to categorical response groups such as MR4.0 can obscure important differences in the distribution of molecular responses in groups of patients from different studies. The definition of UMRD or complete molecular remission (CMR) is entirely dependent on qRT-PCR sensitivity and is not consistent across studies. There is also insufficient data about treatment cessation in patients with atypical *BCR-ABL1* transcripts, which may be associated with different natural histories than that with standard b2a2/b3a2 transcripts, varying from favorable in the case of e19a2 [34] to an adverse outcome with e1a2 [35, 36]. Collectively, the heterogeneity of trial design, limitations, and results makes comparisons across trials particularly perilous.

## Predictive factors

**Fig. 1:**
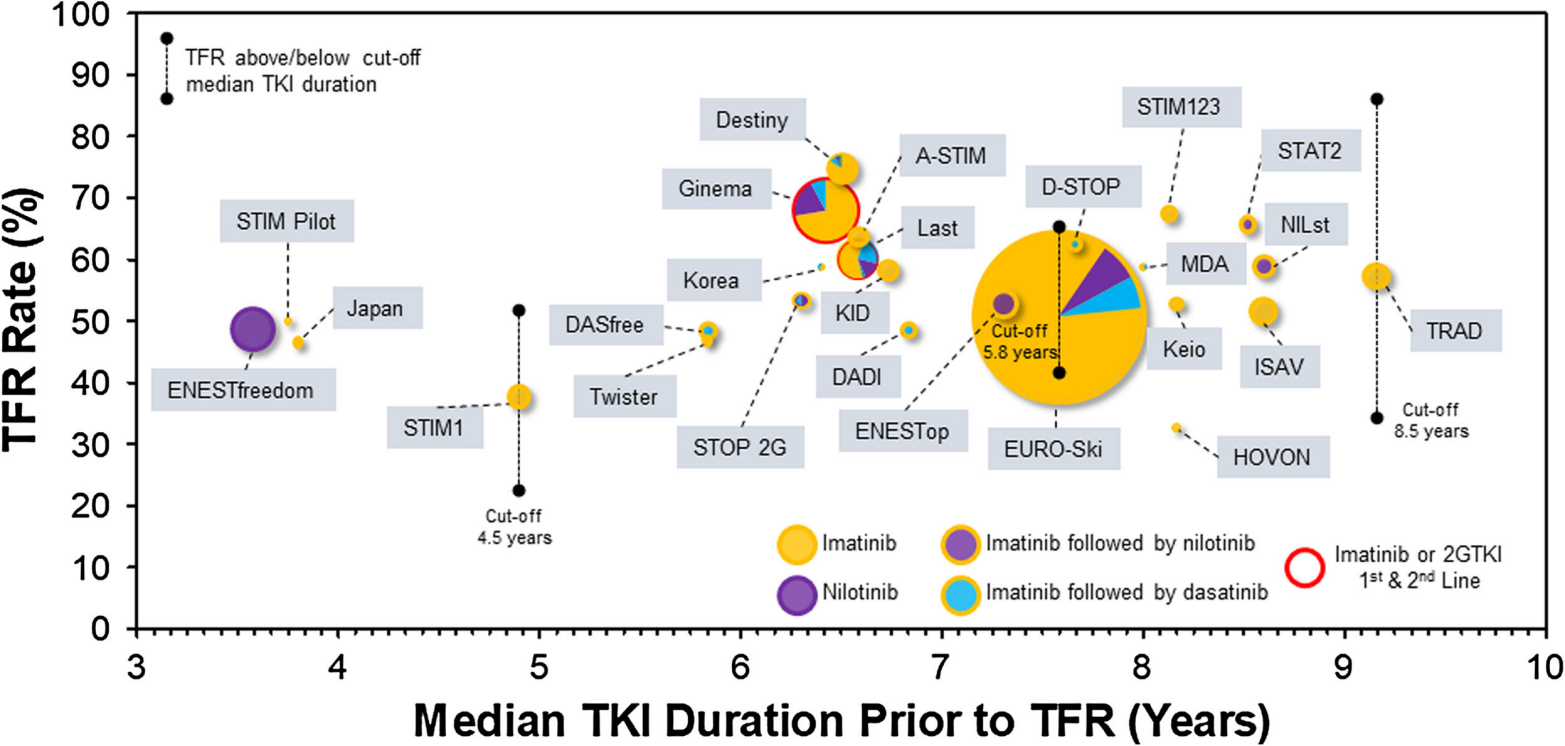
Bubble plot showing the rate of treatment-free remission TFR (%) versus the median TKI duration (years) prior to TKI cessation for different studies listed in Table 1. The size of the circles is proportional to the number of patients in the various studies, and TKI exposure is shown in the color legend. The rate of TFR observed in patients above or below a “cutoff” median duration of TKI therapy is shown for the STIM1, EURO-Ski, and TRAD studies.

A large number of predictive factors have been explored including age, gender, pre-TKI interferon treatment, *BCR-ABL1* transcript (b2a2 versus b3a2), specific TKIs, clinical prognostic scores, early molecular response (EMR), time to DMR, TKI resistance, depth of DMR, duration of DMR, total TKI exposure, comorbidities, functional status, TKI withdrawal syndrome (TWS), NK cell numbers, and other measures of host immunity. Total duration of TKI therapy is perhaps the most consistently reported predictive factor for achieving a TFR. The rate of TFR below and above a duration of TKI cutoff of 4.5 years in STIM1 was 22 versus 50%, 34 versus 57% with a cutoff of 5.8 years in EURO-Ski, and 34.6 versus 80.5% with a cutoff of 8.7 years in the first phase of the TRAD study respectively. Moreover, patients who fail a first TFR attempt may still succeed later following retreatment and further exposure to TKIs. In the RE-STIM study, patients who failed a first TFR and returned to a state of UMRD4.5 (median duration 2.1 years) on re-treatment had a 35% rate of second TFR at 3 years, and up to 72% at 2 years in the subgroup that re-established a DMR within 3 months of the re-instatement of TKI therapy [37]. In contrast to the very large within-study effect of the duration of TKI exposure on the rate of TFR reported, it is striking how comparable, between studies, rates of TFR are over many years of TKI exposure, as shown in Fig. 1. This suggests that other study-related and biological factors must contribute to the success or failure of achieving a TFR and should caution against generalizing the predictive value of any one factor.

While many studies report that deeper molecular responses predict for a greater success of TFR [8, 9, 18], no significant differences were observed between patients in MR4.0 versus MR4.5 versus MR5.0 in an interim analysis of EURO-Ski [38], the largest study to date. Surprisingly, de-escalation of imatinib for 1 year in the Destiny trial [29] prior to discontinuation in patients in either MMR but not MR4.0 or in MR4.0 still resulted in a relapse-free survival (RFS) of 39% in the MMR group while the rate of TFR was 77% in the MR4.0 group at 1 year [15]. The duration of DMR has also been reported to be an important factor in several studies. Each additional year of MR4.0 in EURO-Ski increased the odds of remaining in MMR by 6 months by 13% [21]. Similarly, in the Canadian TRAD study, the rate of TFR at 6 months increased from 41, to 70.4, and to 94.4%, with increasing durations of MR4.0 on imatinib ranging from ≤ 7.8, 7.8 to 10.6, and ≥ 10.6 years respectively [18]. The duration of DMR however is confounded by the duration of TKI exposure as both increase over time. A recent analysis of EURO-Ski has shown that it is the duration of DMR that is more important when adjusting for the duration of TKI treatment [39]. This result might simply reflect a more rapid molecular response since this would predict for a longer period of DMR for any given duration of TKI exposure. The rates of molecular response and early EMR in turn are indicators of TKI sensitivity and these have already been shown to be good predictors for achieving a DMR as well as TFR in some studies [13, 26].

Prior TKI resistance defined by ELN criteria is a strong predictor of TFR failure as shown in both the DADI and STOP 2G-TKI trials suggesting that this group of patients should be excluded from TKI cessation in routine clinical practice [7, 10]. In the STOP 2G-TKI trial, the loss of MMR was 81.8 versus 17.8% in patients who lost MR4.5 in a 3-month landmark analysis versus those who maintained MR4.5 respectively, suggesting that retreatment in the former should probably be considered without further delay [10]. Higher circulating NK cell numbers and other immunological parameters also significantly correlate with improved rates of TFR [7, 40, 41]. While many believe that this is a causal relationship, there is still no direct evidence to support this claim. Moreover, it is becoming clear that CML patients have a number of immune effector defects at diagnosis that tend to normalize with deepening molecular responses on TKI therapy raising an alternative possibility, that it is the elimination of leukemic cells that permits immunological recovery rather than recovery of immunological function directly impacting the molecular response [40]. Several other predictive factors have been reported with less consistency including inferior rates of TFR associated with younger age [9, 26], higher Sokal scores, and better outcomes for patients with prior exposure to interferon [42].

## Imatinib versus second-generation TKIs

Second-generation TKIs induce faster and deeper responses than imatinib. At 5 years, MR4.5 was 54 versus 31% in favor of nilotinib in the standard dose arm compared to imatinib in the ENESTnd trial (1.74-fold increase) [43], and 42 versus 33% in favor of dasatinib compared to imatinib in the DASISION trial (1.27-fold increase) [44]. While this clearly has the potential to increase the number of patients who reach a stable DMR conducive to TKI cessation, it remains unclear if this necessarily increases the rate of TFR. Cessation of first-line nilotinib in the ENESTfreedom study resulted in TFR rate of 48.9% at 1.85 years. While this is not very different from that observed in many imatinib stop trials, ENESTfreedom is an outlier (Fig. 1) in having achieved this degree of TFR after only a median of 3.58 years of TKI exposure. In contrast, trials that included patients who had switched to or were consolidated with a second-generation TKI before stopping do not clearly demonstrate a superior rate of TFR compared to imatinib only stop trials [7, 10, 16, 17, 23, 24] (Fig. 1). However, the latter trials included some patients who had been switched to a second-generation TKI for resistance, a known adverse factor for achieving a TFR. It may be difficult to resolve this issue without a randomized trial by either recruiting patients at the end of a randomized trial of first-line imatinib versus a second-generation TKI into a second stop trial or randomization of first-line imatinib patients to consolidation with a second-generation TKI versus continuation of imatinib until TKI cessation.

## Molecular kinetics of TKI cessation and mechanisms of TFR

The mechanism(s) responsible for successful TKI cessation, whether intrinsic to leukemic stem cells (LSCs), host factors, or likely a combination of both, remains poorly understood. The identification of biomarkers directly linked to such mechanisms may be the only path to improving prediction of TFR success or failure in individual patients. The near binary molecular response kinetics after TKI cessation where there is either a rapid increase of *BCR-ABL1* with loss of TFR, mostly in the first few months, versus stable TFR is unique and remains unexplained. Successful TKI cessation clearly does not rely on the elimination of the leukemic clone as *BCR-ABL1*-positive cells have been shown to persist by sensitive DNA PCR in patients in TFR with UMRD by qRT-PCR [45]. Even more instructive are patients with UMRD who become molecularly positive after stopping TKIs but continue to express *BCR-ABL1* at low levels without further progression and loss of TFR [6]. The latter reveals a loss of competitive repopulation by the leukemic clone relative to normal hematopoietic cells and a change from the conditions that allowed the leukemic clone to dominate earlier at diagnosis.

It has been proposed that TKI exposure sufficient to achieve a DMR selects for leukemic initiating cells (LICs) with variably attenuated growth kinetics [46]. The LICs of patients who relapse early being less impaired than those that relapse later, while those achieving stable TFR might still harbor quiescent LSCs but have no remaining LICs. What is less clear is why the growth kinetics of LICs should be restricted to such a narrow range to explain the short interval in which loss of TFR occurs compared to the wide range observed in survivors of the Hiroshima atomic bomb where an increase in the incidence of CML persisted for well over a decade [47]. Often overlooked is the fitness of non-leukemic HSCs. Although the suppression of normal hematopoiesis by TKIs is generally mild, their potential effect on the fitness of normal HSCs for competitive repopulation is unknown. The appearance of higher TFR success following a period of TKI dose de-escalation observed in the Destiny trial is intriguing and raises the possibility that, in addition to selecting for patients with possibly reduced LSC growth kinetics by excluding patients who fail to remain in MMR before discontinuation, normal HSCs may need time to recover fully from TKI-mediated suppression to be able to compete successfully with LSCs. Other host factors, including the previously mentioned restoration of immunological defects with DMR, may be just as important as intrinsic properties of normal HSC and LSCs. It is increasingly appreciated that leukemic cells can reprogram the bone marrow microenvironment to favor LSC growth and that the reversibility of microenvironmental changes to normal following treatment-induced reduction of leukemic cells may play an important role in determining the risk of leukemia relapse [48–51].

## Clinical practice guidelines for TKI cessation

Given the heterogeneity of clinical trials and results, and the difficulty of accurately predicting the success of TFR in individual patients, it is not surprising that recommendations for attempting TFR in clinical practice vary. Published criteria for attempting TFR outside of clinical trials include expert opinion [52] as well as formal guidelines from ESMO [53] and NCCN [2] as shown in Table 2. An update of the ELN guidelines should be forthcoming in the near future. There is general emphasis on ensuring patient safety by restricting discontinuation to CML-CP patients with no prior history of advanced disease and frequent monitoring with a sensitive (≤ MR4.5) qRT-PCR standardized to the International Scale with a rapid turnaround time of 2 to 4 weeks. Beyond that, there are significant differences for the minimal duration of TKI exposure ranging from 3 (NCCN) to 8 years (Hughes et al.), for DMR of ≤ MR4.0 (NCCN) or ≤ MR4.5 (Hughes et al., ESMO) for a duration of at least 2 years, and for retreatment with loss of MMR (NCCN) or not defined (Hughes et al., ESMO). Similarly, ideal candidates are recognized as non-high Sokal (Hughes et al., ESMO, not stipulated in NCCN), with an optimal response (Hughes et al., ESMO), or simply with no prior TKI resistance (NCCN). While Hughes et al. caution against discontinuation in patients with atypical transcripts, the ESMO and NCCN guidelines only stipulate that transcripts be measurable.

**Table 2:** Criteria for stopping TKIs from expert recommendations and guidelines

Criteria	Hughes* [52]			NCCN** [2]	ESMO** [53]
	Green	Yellow	Red		
CML past history	CP only	Resistance or KD mutation	AP/BP	CP only	CP only
Sokal	Non-high	High	NA		Non-high
Response to TKI therapy	Optimal	Warning	Failure	No resistance	Optimal
*BCR-ABL1* transcript	Typical	Quantifiable atypical	Not quantifiable	Measurable	Measurable
Duration TKI	≥ 8 years	3–8 years	< 3 years	≥ 3 years	≥ 5 years
DMR	≤ MR4.5	≤ MR4.0	> MR4.0	≤ MR4.0	≤ MR4.5
Duration DMR	≥ 2 years	1–2 years	< 1 year	≥ 2 years	≤ MR4 ≥ 2 years
Retreatment				Loss MMR	
PCR sensitivity	≤ MR4.5				≤ MR4.5
Frequency of monitoring	Q1M 1st 6 months, Q2–3 months			Q1M × 6, Q6W × 6M, Q3M	Q1M × 6, Q6W × 6, Q3M
PCR result turnaround time	≤ 4 weeks			≤ 2 weeks	

*M* months, *W* weeks

*Expert recommendations

**Guidelines

## Conclusion and future directions

The emphasis on defining minimal criteria for discontinuation TKI therapy in clinical practice overlooks what rate of TFR should be considered acceptable, as imprecise as it is to establish this in individual patients and, more importantly, the competing patient-specific justification for TKI cessation. Patients on TKIs in DMR have no significant risk of disease-related death. On the other hand, stopping TKIs appears to be safe while continued TKI therapy may expose some patients to significant treatment-related morbidity and mortality that could justify to attempt TKI discontinuation when there is lower probability of success. For instance, the cumulative risk of a cardiovascular event (CVE) in some patients treated with dasatinib or nilotinib may far outweigh the inconvenience and risk of a failed TFR attempt. By definition, patients with intermediate and high Framingham risk scores have a risk of a CVE event over 10 years of 10–20% and greater than 20% respectively. This already important risk is increased 3-fold and nearly 6-fold respectively in patients taking standard dose nilotinib [43]. One could consider dose de-escalation alone to mitigate the risk of CVEs in such patients [29, 54], switch to a TKI with less risk of a CVE such as bosutinib [55] or even imatinib [56], or simply attempt TKI cessation. Patients may have equally compelling reasons to want to stop TKI therapy due to chronic intolerance, pregnancy, or other personal reasons and may similarly accept to attempt TKI cessation with less than optimal conditions.

In contrast, there may be much less medical justification to attempt discontinuation in patients in DMR on imatinib, who require minimal monitoring every 6 months, are asymptomatic, and have no financial or personal incentive to stop therapy. Some patients in DMR or with UMRD may also have different degrees of comfort with delaying retreatment until MMR is lost and wish to restart TKI treatment earlier. Even if the probability of achieving a stable TFR could be defined more precisely, any recommendation to stop TKI therapy should remain sufficiently flexible to accommodate competing medical risks and patient acceptance. It is particularly important to ensure that TKI cessation not become a standard for continued medical coverage by third-party payers. These considerations caution against the development of overly rigid guidelines on when to stop TKI therapy and reinforce that stopping TKIs truly belongs in the realm of personalized medicine.

At this juncture in time, there is not much more to gain from conducting additional non-randomized prospective studies of TKI cessation. Attention should increasingly turn to prospective randomized trials including new agents alone or in combination with TKIs and basic/translational studies to elucidate the mechanisms and the discovery of related biomarkers that determine the success or failure of TFR.
